# New formulation of a recombinant anthrax vaccine stabilised with structurally modified plant viruses

**DOI:** 10.3389/fmicb.2022.1003969

**Published:** 2022-09-09

**Authors:** Dmitriy L. Granovskiy, Ekaterina M. Ryabchevskaya, Ekaterina A. Evtushenko, Olga A. Kondakova, Marina V. Arkhipenko, Tatiana B. Kravchenko, Irina V. Bakhteeva, Vitalii S. Timofeev, Nikolai A. Nikitin, Olga V. Karpova

**Affiliations:** ^1^Department of Virology, Faculty of Biology, Lomonosov Moscow State University, Moscow, Russia; ^2^Federal Budget Institution of Science State Research Center for Applied Microbiology and Biotechnology (FBIS SRCAMB), Obolensk, Russia

**Keywords:** anthrax, protective antigen (PA), vaccine candidate, plant virus, adjuvant, tobacco mosaic virus, structurally modified plant virus

## Abstract

Anthrax is a disease caused by *Bacillus anthracis*. The most promising approach to the development of anthrax vaccine is use of the anthrax protective antigen (PA). At the same time, recombinant PA is a very unstable protein. Previously, the authors have designed a stable modified recombinant anthrax protective antigen with inactivated proteolytic sites and substituted deamidation sites (rPA83m). As a second approach to recombinant PA stabilisation, plant virus spherical particles (SPs) were used as a stabiliser. The combination of these two approaches was shown to be the most effective. Here, the authors report the results of a detailed study of the stability, immunogenicity and protectiveness of rPA83m + SPs compositions. These compositions were shown to be stable, provided high anti-rPA83m antibody titres in guinea pigs and were able to protect them from a fully virulent 81/1 *Bacillus anthracis* strain. Given these facts, the formulation of rPA83m + SPs compositions is considered to be a prospective anthrax vaccine candidate.

## Introduction

Anthrax is a severe disease caused by a gram-positive spore-forming bacterium *Bacillus anthracis.* Anthrax remains a menace. Thawing of permafrost leads to local disease outbreaks since *B. anthracis* spores are extremely resilient and are able to persist in the soil for a long period of time. In 2016, more than 36 people were exposed to anthrax infection on Yamal peninsula, one human death was documented and some two thousand reindeers died. Soil analyses performed in 2015 in Yakutia revealed three different anthrax strains (Goudarzi, 2016; Timofeev et al., 2019). Moreover, there is a threat of anthrax-related bioterror attacks like the one that took place in United States in 2001 (Centers for Disease Control and Prevention, 2001; Inglesby et al., 2002). Given these facts, it is desirable to closely monitor potential anthrax accidents and keep them under control. The most efficient way to prevent anthrax is vaccination.

Currently, licenced anthrax vaccines are represented by two attenuated vaccines (Russian vaccine based on the STI-1 strain and a Chinese vaccine based on the A16R strain) and vaccine formulations based on non-encapsulated *B. anthracis* strains cell filtrates—Anthrax Vaccine Adsorbed (AVA, Biothrax*™*, Lansing, MI, United States) and Anthrax Vaccine Precipitated (AVP, United Kingdom). The use of all existing vaccines is associated with a high level of adverse reactions (World Health Organization, 2012). Cell filtrate-based vaccines are the most widely used for anthrax prevention in the world. For this type of vaccine, adverse reactions might be due to the presence of an indeterminate amount of anthrax oedema factor and lethal factor along with other bacterial components, while the contribution of these to protective immunity is not generally accepted (Modi et al., 2021). Thus, the development of a new approach to anthrax vaccine creation is considered to be desirable.

Most modern anthrax vaccines and vaccine candidates target anthrax toxin (Kondakova et al., 2021). Anthrax toxin consists of protective antigen (PA), oedema factor (EF), and lethal factor (LF). During infection, PA acts as transport for other toxin components (EF and LF) through the cell barriers. Thus, in the absence of EF and LF, PA is totally harmless for mammal recipients. At the same time, PA-induced immune response is sufficient for disease prevention (Little et al., 1997; Clement et al., 2010). Therefore, recombinant PA (rPA) is the most promising basis for safe and effective anthrax vaccine. However, the stability of rPA is very low and decreases further when rPA is adsorbed to aluminium hydroxide, the adjuvant used in AVA and in almost all anthrax vaccine candidates in clinical trials (Kondakova et al., 2019). These features of PA imply the need for research into ways of stabilising PA and for an alternative carrier for an anthrax vaccine.

Previously, the authors developed a modified rPA with an inactivated furin-sensitive site (^162^NSRKKR^167^ was substituted with ^162^QSSNKE^167^), an inactivated chymotrypsin-sensitive site (^313^FF^314^ was deleted) and deamidation-prone Asn^713^ and Asn^719^ replaced by glutamines (Ryabchevskaya et al., 2022). Modified rPA was named rPA83m. rPA83m was demonstrated to be considerably more stable than non-modified rPA while maintaining antigenic properties after storage. In the same study, the authors suggested spherical particles (SPs) obtained from tobacco mosaic virus through its thermally-induced structural rearrangement as a carrier and a stabiliser for rPA83m. Using plant viruses as carriers in vaccine formulations is a common approach for vaccine development (Denis et al., 2008; Trifonova et al., 2017; Kovalenko et al., 2022). SPs are known to be safe for mammals and biodegradable in the recipient’s organism (Bruckman et al., 2014; Nikitin et al., 2018a,b). rPA83m has been shown to be more stable as a part of rPA83m + SPs compositions than in the form of individual protein. The antigenic properties of rPA83m as a part of compositions with SPs remained unchanged even after incubation at + 37°C for 40 days (Ryabchevskaya et al., 2022). Thus, SPs were accepted to be a convenient stabiliser and carrier for rPA83m.

In the current study, rPA83m + SPs compositions were evaluated as an anthrax vaccine candidate. A detailed stability experiment was carried out, which demonstrated that SPs significantly decrease the rPA83m degradation rate. The study features the immunogenicity and protectiveness evaluation of rPA83m + SPs compositions. rPA83m + SPs compositions were shown to induce the immune response in guinea pigs. Moreover, the immunogenic properties of rPA83m in these compositions remained unchanged after 27 days of incubation at + 37°C, in contrast to rPA83m adsorbed to aluminium hydroxide: in the latter case induced anti-rPA83m antibody titres significantly decreased for stored samples. Immunisation with rPA83m + SPs compositions provided up to 100% protection against the spore challenge of fully virulent *B. anthracis* 81/1 strain (pXO1^+^, pXO2^+^). Animals immunised with rPA83m + SPs compositions were significantly more resilient to the infection than animals immunised with rPA83m adsorbed to aluminium hydroxide. Furthermore, the protectiveness of rPA83m + SPs compositions did not significantly decrease for samples stored for 27 days at + 37°C. Current data show that using rPA83m + SPs compositions is a promising approach to anthrax vaccine development. This approach can be a solution to major problems associated with existing PA-based vaccines.

## Materials and methods

### Recombinant antigen rPA83m production

The pQE-rPA83m plasmid vector, constructed previously by the authors (Ryabchevskaya et al., 2022), was employed for rPA83m production. The expression and purification procedures were conducted according to Ryabchevskaya et al. (2022). rPA83m was dialysed against Milli-Q (Simplicity UV, Merck Millipore, Darmstadt, Germany) in the ratio of 1:250 for 4 h with hourly water changing and followed by sterilisation using 0.2 μm filters [CHROMAFIL^§^ CA-20/25(S), 729024]. The concentration of rPA83m was determined using spectrophotometry (NanoDrop 2000 spectrophotometer, Thermo Scientific, Waltham, MA, United States) at 280 nm (E_280*nm*_ 0.1% = 0.954). The extinction coefficient (E_0_._1%_) of recombinant protein was calculated using PROTPARAM from the Expert Protein Analysis System (EXPaSy) proteomics server of the Swiss Institute of Bioinformatics (Gasteiger et al., 2003). Endotoxin level of rPA83m was determined using the endpoint chromogenic LAL assay (Hycult Biotech Inc., Wayne, PA, United States) as described previously (Kovalenko et al., 2022) and was 2.31 EU/dose.

### Spherical particles formation

Isolation, purification of TMV and the generation of SPs was carried out according to Trifonova et al. (2015) with some modifications. For SPs production TMV with a concentration of 2 mg/ml was aliquoted into 500μl in standard 1.5 mL polypropylene tubes (Greiner Bio-One GmbH, Frickenhausen, Germany) and incubated at 98°C in “Termite” thermostat (DNA technology, Moscow, Russia) for 10 min. Endotoxin level of SPs was determined in the same way as of rPA83m and was 0.183 EU/dose.

### rPA83m stability evaluation in individual formulation and in compositions with spherical particles

Samples of individually formulated rPA83m and rPA83m in compositions with SPs (rPA83m + SPs) were incubated at +4, +25, or +37°C. Each sample contained 1 μg of rPA83m with the concentration of 0.1 μg/μl. rPA83m/SPs mass ratio within compositions was 1/10. Therefore, rPA83m + SPs samples contained 1 μg of rPA83m and 10 μg of SPs. All samples were prepared in PBS. The volume of samples was 10 μl. The experiment was performed with five replicates. All samples for all replicates were prepared simultaneously, after which a part of samples were immediately frozen. These samples were further used as non-incubated control standards (“day 0” checkpoint). Other samples were subjected to incubation at +4,25, or +37°C. At each checkpoint (for samples incubated at +4°C—every 14 days, at +25°C—every seven or 12 days, depending on the experiment, at +37°C—every 3 days), five samples of individually formulated rPA83m and five samples of rPA83m + SPs were frozen and stored at -70°C. The examination of rPA83m state was performed separately at each checkpoint by protein electrophoresis in 8–20% SDS-PAGE with Coomassie G-250 staining. Immediately before the electrophoresis analysis, frozen samples were thawed, mixed with 10 μl of sample load buffer [0.125M Tris-HCl, 15% glycerol (v/v), 0.05% β-mercaptoethanol (v/v), 0.0226% SDS (w/v), 0.0004% bromphenol blue (w/v)] and heated at 95°C for 5 min. The whole sample volume was loaded into a corresponding gel slot. At each checkpoint, samples of individually formulated rPA83m (five replicates) and rPA83m + SPs (five replicates) were loaded on the gel together with non-incubated control standards of both individually formulated rPA83m (two replicates) and PA83m + SPs (two replicates).

For the analysis of rPA83m stability the relative percentage of non-degraded rPA83m within incubated samples was determined. The major rPA83m bands within non-incubated control standards were used as relative bands (100%) for the calculation of non-degraded rPA83m quantity within corresponding incubated samples. Control standards of individually formulated PA83m and PA83m + SPs were used for the analysis of incubated samples of individually formulated PA83m and SPs + PA83m, respectively. For each sample, the mean of two values obtained by its comparison with each of two corresponding standards was taken for the remaining percentage of non-degraded rPA83m. Mean values of all replicates for each checkpoint were used for the graphical visualisation and pairwise comparison of groups of replicates of individually formulated PA83m and PA83m + SPs. The calculation was performed using ChemiDoc*™* XRS + System with Image Lab*™* Software (Bio-Rad Laboratories, Hercules, CA, United States). All sporadic deviations from the established method are marked in the descriptions of corresponding figures and did not disrupt the statistical analysis.

### Ethical statement

The methods used in experiments with guinea pigs were performed in accordance with relevant guidelines and regulations and approved by the State Research Center for Applied Microbiology and Biotechnology Bioethics Committee (Permit No: VP 2021/4 dated 16.06.2021).

### Immunisation of guinea pigs

Agouti guinea pigs (6–8 weeks old, 275 ± 25 g) of both genders were used in the experiments. Animals were purchased from the breeding centre “Andreevka” of the “Scientific Center of Biomedical Technology” of the Federal Medical-Biological Agency of Russia. The study included six experimental groups of ten animals (5 + 5) each. Animals of groups 1 and 4 were immunised with rPA83m (30 μg per animal); of groups 2 and 5 with SPs + rPA83m compositions (300 μg SPs + 30 μg rPA83m per animal); of groups 3 and 6 with rPA83m with Al(OH)_3_ (FSE “Shchelkovo biocombinat”, Moscow region, Russia) [30 μg rPA83m + 300 μg Al(OH)_3_ per animal]. Animals from groups 1, 2, and 3 were immunised with non-incubated formulations, which were frozen immediately after preparation and stored at -20°C. Animals from groups 4, 5, and 6 were immunised with formulations that were incubated for 27 days at + 37°C before being frozen at -20°C. The non-immune control was represented by a group of five animals (3 + 2) immunised only with SPs (300 μg per animal) (group 7) and a group of five animals (2 + 3) immunised with PBS (group 8). All administered samples were prepared in PBS; the final volume was 0.3 ml. Before immunisation all formulations were thawed and thoroughly mixed immediately before injection. Animals were immunised twice, at 28-day intervals. Animals were vaccinated subcutaneously in the inner part of the upper thigh. Blood was collected from the marginal ear vein 20 days after the second immunisation. The scheme of the study is presented in the Section “Results” (Figure 4).

### Enzyme-linked immunosorbent assay

ELISA was performed according to the protocol described in Kovalenko et al. (2022). Ninety six-well microplates (Greiner Bio-One 655001, Frickenhausen, Germany) were coated with 10 μg/ml of rPA83m or SPs. Secondary antibodies to guinea pig total IgG conjugated with HRP (Jasckson Immunoresearch 706-035-148, West Grove, PA, United States) were used in a dilution of 1/10,000. Measurement of antibody levels was performed with at least three replicates; the individual sera titres represented are the geometric means of all analytical repeats.

### *Bacillus anthracis* spore challenge

*Bacillus anthracis* 81/1 strain (pXO1^+^, pXO2^+^), deposited in the SRCAMB collection, was used to infect animals. At 21 days after the second immunisation, all guinea pigs were subcutaneously challenged with 2,500 spores per animal. At 20 days after the first challenge all survived guinea pigs (group 1—all, group 2—all, group 3—8, group 4—all, group 5—9, group 6—9, group 7—4, group 8—3) were subjected to the second challenge with the same strain in a ten-fold higher dose (25,000 spores per animal). The scheme of the study is presented in the Section “Results” (Figure 4). The animals were observed for 21 days after reinfection; the surviving animals were humanely euthanised by CO_2_ inhalation.

### Statistical analysis

In stability studies the Wilcoxon-Mann-Whitney Test was used to examine differences between two groups. In immunogenicity studies the Wilcoxon-Mann-Whitney Test and the Kruskal-Wallis test with a *post hoc* Dunn’s test was used for pairwise and multiple comparisons, respectively. The statistical tests used in each individual experiment have been indicated in figure captions. The *F*-test was used to compare variances. Probability values (*p*-values) of less than 0.05 were considered to be significant. For comparison of the survival rate between different groups, the Gehan-Breslow-Wilcoxon test was used and the Holm-Bonferroni method was applied for adjusting p-values for multiple comparisons. The statistical processing of the results and graph plotting were carried out using GraphPadPrism 9.1.0 (GraphPad Software, La Jolla, San Diego, CA, United States).

## Results

### The impact of spherical particles on rPA83m stability

**FIGURE 1:**
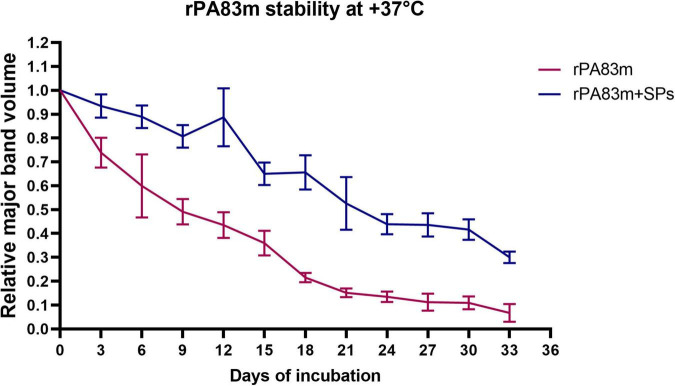
Stability of individually formulated rPA83m and rPA83m as a part of rPA83m + SPs compositions at +37°C. Individually formulated rPA83m and rPA83m + SPs compositions were incubated at +37°C in PBS. In both cases, rPA83m concentration was 1 μg/μl. rPA83m/SPs mass ratio was 1:10. For each checkpoint, relative major band volume means are presented for both individually formulated rPA83m (red) and rPA83m as a part of rPA83m + SPs compositions (blue) (the number of replicates was *n* = 5). Stability evaluation for all incubated replicates was carried out by 8–20% SDS-PAGE analysis. For each replicate, the mean value of major band volumes determined by comparison with two corresponding non-incubated samples was used for graph plotting and group comparison. Bars represent 95% CI.

An evaluation of the stability of individually formulated rPA83m and rPA83m + SPs compositions was carried out. The stability experiments were performed at +37°C (in order to model rapid protein ageing), at +25°C (room temperature representing a disruption in the cold chain storage of a vaccine) and at +4°C (a common vaccine storage temperature). Stability at +37°C was examined for 33 days at 3-days intervals (Figure 1). At each checkpoint the relative volume of rPA83m major band on the SDS-PAGE gel was significantly higher in rPA83m + SPs samples than in individually formulated rPA83m samples (*p*-value < 0.05).

**FIGURE 2:**
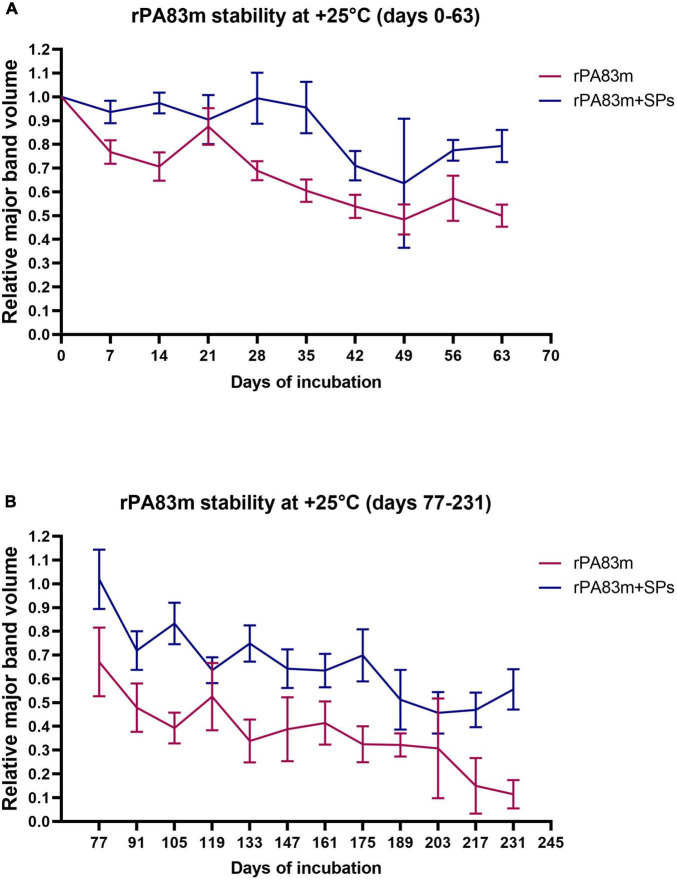
Stability of individually formulated rPA83m and rPA83m as a part of rPA83m + SPs compositions at +25°C. Two independent stability evaluation experiments were carried out: for days 0–63 **(A)** and for days 77–231 **(B)**. In each experiment individually formulated rPA83m and rPA83m + SPs compositions were incubated at +25°C in PBS. In both cases, rPA83m concentration was 1 μg/μl. rPA83m/SPs mass ratio was 1:10. For each checkpoint, relative major band volume means are presented for both individually formulated rPA83m (red) and rPA83m as a part of rPA83m + SPs compositions (blue) (the number of replicates was *n* = 5 except for rPA83m days 21, 91, 175, and 203 and rPA83m + SPs day 175, in which cases it was *n* = 4, 4, 3, 3, and 4, respectively). Stability evaluation for all incubated replicates was carried out by 8–20% SDS-PAGE analysis. For each replicate, the mean value of major band volumes determined by comparison with two corresponding non-incubated samples was used for graph plotting and group comparison. Bars represent 95% CI.

Stability at +25°C was initially examined for 63 days at 7-day intervals (Figure 2A). After the analysis of data obtained on initial stability, an additional experiment was carried out at the same temperature, since the level of protein degradation achieved at day 63 was considered to be insufficient. The second experiment at + 25°C lasted 231 days; the samples were analysed at 14-day intervals from day 77 (Figure 2B). At each checkpoint in both experiments at +25°C, except for days 21, 49, 119, and 203, the relative volume of rPA83m major band was significantly higher in rPA83m + SPs samples than in individually formulated rPA83m samples (*p*-value < 0.05). The lack of significance in the difference between the two variants of rPA83m formulations at days 21, 49 119, and 203 might have been due to some methodological errors that occurred during the preparation of individually formulated rPA83m samples for SDS-PAGE analysis. Thus, the stability of rPA83m as a part of rPA83m + SPs compositions was shown to be significantly higher than the stability of individually formulated rPA83m both at +37°C and at +25°C.

**FIGURE 3:**
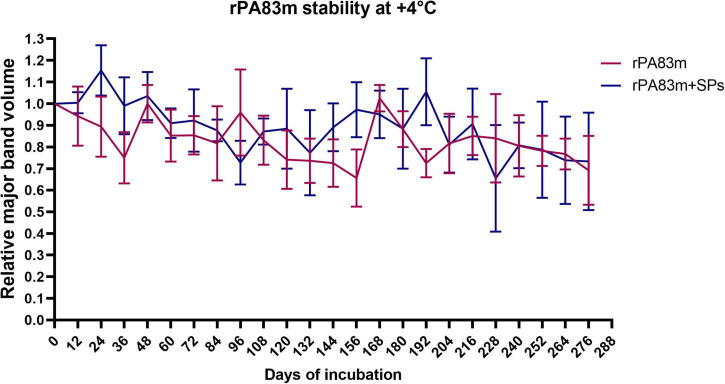
Stability of individually formulated rPA83m and rPA83m as a part of rPA83m + SPs compositions at +4°C. Individually formulated rPA83m and rPA83m + SPs compositions were incubated at +25°C. In both cases rPA83m concentration was 1 μg/μl. rPA83m/SPs mass ratio was 1:10. For each checkpoint, relative major band volume means are presented for both individually formulated rPA83m (red) and rPA83m as a part of rPA83m + SPs compositions (blue) (the number of replicates was *n* = 5, except for rPA83m day 72 and rPA83m + SPs day 264, in which case it was *n* = 4 and 3, respectively). Stability evaluation for all incubated replicates was carried out by 8–20% SDS-PAGE analysis. For each replicate, the mean value of major band volumes determined by comparison with two corresponding non-incubated samples (except for rPA83m + SPs at day 228, in which case only 1 standard sample was used) was used for graph plotting and group comparison. Bars represent 95% CI.

Stability at +4°C was examined for 276 days at 12-days intervals (Figure 3). The analysis of the data obtained revealed no differences between the stability of rPA83m in rPA83m + SPs compositions and individually formulated rPA83m. That might indicate that a 276-day incubation is not enough to reveal the stabilising properties of SPs at +4°C. However, the fact that the relative volume of rPA83m major band did not fall below 0.6, either in rPA83m + SPs compositions samples or individually formulated rPA83m samples, indicates the overall high stability of these formulations at a common vaccine storage temperature.

### Vaccine candidate immunogenicity

**FIGURE 4:**
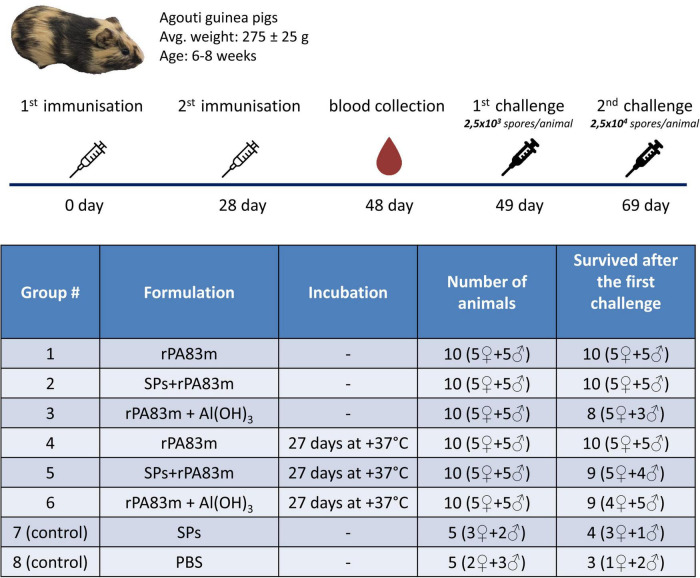
Immunisation schedule and description of guinea pig groups involved in the experiment to evaluate the immunogenicity and the protectiveness of various rPA83m formulations. Groups of guinea pigs were immunised subcutaneously twice at 28-day intervals with a corresponding formulation containing 30 μg of rPA83m. In rPA83m + SPs formulation rPA83m/SPs mass ratio was 1:10. In rPA83m + Al(OH)_3_ formulation rPA83m/Al(OH)_3_ mass ratio was 1:10. Animals in group 7 (control) were immunised with 300 μg of SPs. Animals in group 8 (control) were immunised only with PBS. Incubated samples were subjected to incubation at +37°C for 27 days. All administered samples were prepared in PBS; the final volume was 0.3 ml. Blood was collected from the marginal ear vein 20 days after the second immunisation. At 21 days after the second immunisation, all guinea pigs were subcutaneously challenged with *B. anthracis* strain 81/1 2,500 spores per animal. At 20 days after the first challenge, all surviving guinea pigs were subjected to a second challenge with 25,000 spores of the same strain per animal.

In order to evaluate the rPA83m + SPs compositions’ immunogenicity and its maintenance upon storage, the immunisation of guinea pigs was carried out. For the purposes of examining SPs’ impact on vaccine candidate immunogenicity for both non-incubated and incubated formulations, the immunisation with individual rPA83m and rPA83m + Al(OH)_3_ formulations was also carried out. Six groups of ten guinea pigs were injected with individual rPA83m (groups 1 and 4), rPA83m + SPs (groups 2 and 5) or rPA83m + Al(OH)_3_ (groups 3 and 6) formulations. Animals from groups 1, 2, and 3 were immunised with non-incubated formulations, while animals from groups 4, 5, and 6 were immunised with formulations incubated for 27 days at +37°C. As a control, two groups of five guinea pigs were immunised with SPs alone (group 7) or with PBS solution (group 8). The immunisation schedule and the groups’ description summary are represented in Figure 4.

All animals were observed daily during the immunisation period, and their weight was recorded every 2 days. No animal deaths, nor any signs of abnormality were detected. The dynamic of weight of the guinea pigs in each group in comparison with the control group immunised with PBS (group 8) is represented in Supplementary Figure 1. There was no considerable difference in weight gain between groups 1–7 and group 8.

**FIGURE 5:**
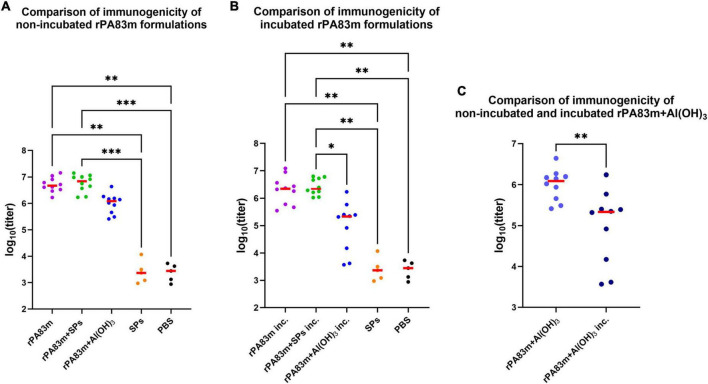
Immunogenicity of rPA83m as a part of various formulations in guinea pigs. **(A)** Comparison of immunogenicity of non-incubated rPA83m formulations. **(B)** Comparison of immunogenicity of incubated rPA83m formulations. **(C)** Comparison of immunogenicity of non-incubated and incubated rPA83m + Al(OH)_3_. Incubated formulations marked with “inc.” were incubated at +37°C for 27 days. 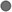, titres of mice; **–**, median. Groups of guinea pigs were immunised subcutaneously twice at 28-day intervals. The scheme of the study is presented in Figure 4. Blood was collected from the marginal ear vein 20 days after the second immunisation. Sera titres were evaluated using indirect ELISA (antigen concentration on microplate—10 μg/ml). *P*-values were calculated using the Kruskal-Wallis test with a *post hoc* Dunn’s test for multiple comparisons **(A,B)** or the Wilcoxon-Mann-Whitney Test for two groups comparison **(C)**. *P < 0.05, ^**^P < 0.01, ^***^P < 0.001. The complete data on anti-rPA83m sera titres for each guinea pigs immunisation group are presented in Supplementary Table 1.

Blood was collected in 20 days after boost, from the marginal ear vein. For all sera samples the levels of anti-rPA83m total IgG were measured (Figure 5). For serum samples collected from animals immunised with non-incubated samples there was no statistically significant difference between anti-rPA83m titres elicited by individual rPA83m (group 1, median titre 4.73 × 10^6^), rPA83m + SPs (group 2, median titre 6.94 × 10^6^) and rPA83m + Al(OH)_3_ (group 3, median titre 1.22 × 10^6^), according to Dunn’s multiple comparison test (Figure 5A and Supplementary Table 1). However, significant differences from the anti-rPA83m titres in the sera from control groups immunised with SPs (group 7, median titre 1.8 × 10^3^) or PBS (group 8, median titre 1.77 × 10^3^) were only detected for groups 1 and 2, and not for group 3.

For incubated formulations of individual rPA83m and rPA83m + SPs the similar results were obtained (Figure 5B and Supplementary Table 1). Anti-rPA83m titres induced by incubated individual rPA83m (group 4, median titre 2.23 × 10^6^) or incubated rPA83m + SPs (group 5, median titre 2.21 × 10^6^) did not differ and were significantly higher than the anti-rPA83m titres detected in negative controls sera from groups 7 and 8. In contrast to the non-incubated samples, the Dunn’s multiple comparison test revealed a significant difference between anti-rPA83m titres induced by incubated rPA83m + Al(OH)_3_ (group 6, median titre 2.16 × 10^5^) and those induced by incubated rPA83m + SPs, which were ten times higher. No significant difference was detected between titres in sera collected from group 6 and control groups 7 or 8.

Pairwise comparison showed no statistically significant change in IgG titres elicited by incubated formulations of individual rPA83m or rPA83m + SPs compared to the corresponding non-incubated formulations. Nevertheless, it is noteworthy that, in the case of immunisation with incubated formulations, anti-rPA83m sera titres induced by rPA83m + SPs were characterised by significantly lower variance than those induced by individual rPA83m (*F*-test *p*-value = 0.0033), while such a difference was not detected for titres elicited by non-incubated formulations. For aluminium-containing formulations, a pairwise comparison revealed a significant decrease in antibody titres elicited by incubated rPA83m + Al(OH)_3_, compared to the non-incubated formulation. The median titre elicited by incubated rPA83m + Al(OH)_3_ was 5.65 times lower than that elicited by non-incubated rPA83m + Al(OH)_3_ formulation.

For groups 2 and 5, which were immunised with rPA83m + SPs compositions, the anti-SPs total IgG titres were also evaluated (Supplementary Table 2). The Dunn’s multiple comparison test revealed no significant difference between anti-SPs titres induced by non-incubated rPA83m + SPs (group 2, median titre 4.63 × 10^4^), incubated rPA83m + SPs (group 5, median titre 2.35 × 10^4^) formulations and SPs alone (group 7, median titre 3.19 × 10^4^). For each of these groups, anti-SPs titres were significantly higher than those in PBS-immunised control group sera (group 8, median titre 5.52 × 10^3^).

**FIGURE 6:**
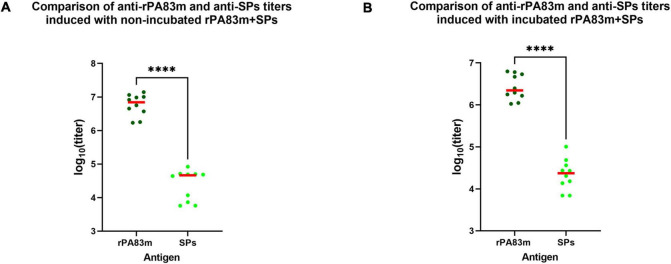
Comparison of anti-rPA83m and anti-SPs immune responses to rPA83m + SPs in guinea pigs. **(A)** Comparison of anti-rPA83m and anti-SPs titres induced with non-incubated rPA83m + SPs. **(B)** Comparison of anti-rPA83m and anti-SPs titres induced with incubated rPA83m + SPs. Incubated formulations were incubated to +37°C for 27 days. 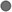, titres of mice; **–**, median. Groups of guinea pigs were immunised subcutaneously twice at 28-day intervals. The scheme of the study is presented in Figure 4. Blood was collected from the marginal ear vein 20 days after the second immunisation. Sera titres were evaluated using indirect ELISA (antigen concentration on microplate—10 μg/ml). *P*-values were calculated using the Wilcoxon-Mann-Whitney Test. ^****^P < 0.0001. The complete data on anti-SPs sera titres for corresponding guinea pig immunisation groups are presented in Supplementary Table 2.

According to the results of pairwise comparison, in groups 2 and 5 anti-SPs titres were significantly lower than anti-rPA83m titres induced by the same formulation (Figure 6). The anti-SPs titres were 150 times and 94 times lower than corresponding anti-rPA83m titres for groups of guinea pigs immunised with non-incubated and incubated rPA83m + SPs, respectively.

### The protectiveness of the vaccine candidate

To evaluate the protectiveness of the vaccine candidate, the influence of SPs on the protectiveness and the ability of the vaccine candidate to maintain protectiveness after storage, all guinea pigs (Figure 4) were infected with *B. anthracis* spores 21 days after the second immunisation. The infection was performed with 2,500 spores of *B. anthracis* 81/1 strain (pXO1^+^, pXO2^+^) per animal. At 20 days after the first challenge, the death rate was low and did not exceed 10% for groups immunised with non-incubated rPA83m formulations, 20% for groups immunised with incubated rPA83m formulations and 40% for control groups (the cumulative data from groups 7 and 8). No statistical differences in the survival rate were detected between any group of guinea pigs immunised with rPA83m formulations and control groups. The survival curves for all groups of animals are presented in Supplementary Figure 2. In this regard, the second spore challenge was performed 20 days after the first one.

**FIGURE 7:**
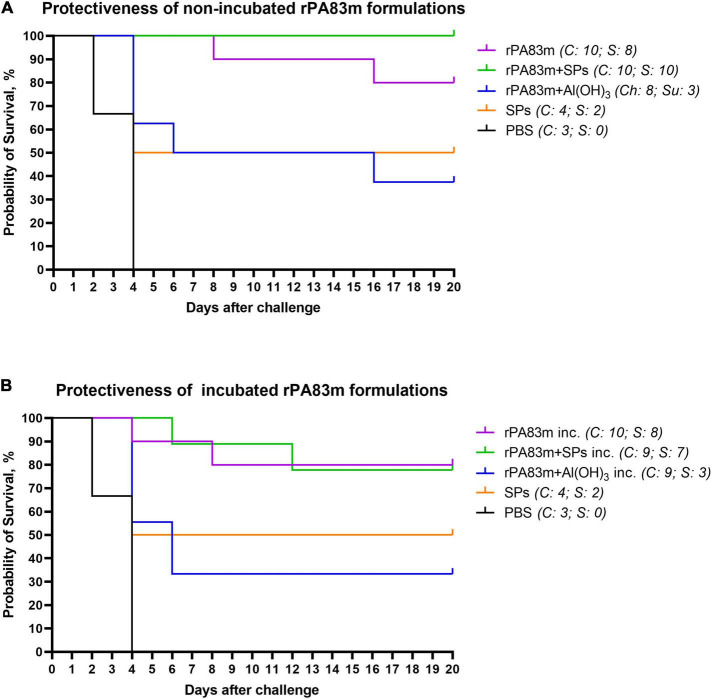
Protectiveness of rPA83m formulations in guinea pigs after second *B. anthracis* strain 81/1 anthrax spore challenge. **(A)** Protectiveness of non-incubated rPA83m formulations. Figure represents survival curves of guinea pig groups immunised with non-incubated formulations of rPA83m (*n* = 10), rPA83m + SPs (*n* = 10), rPA83 + Al(OH)_3_ (*n* = 8), SPs (*n* = 4) or PBS (*n* = 3). **(B)** Protectiveness of non-incubated rPA83m formulations. Figure represents survival curves of guinea pig groups immunised with rPA83m inc. (*n* = 10), rPA83m + SPs inc. (*n* = 9), rPA83 + Al(OH)_3_ inc. (*n* = 9), SPs (*n* = 4), or PBS (*n* = 3). Incubated formulations marked with “inc.” were incubated at +37°C for 27 days. Groups of guinea pigs (*n* = 10 for groups immunised with rPA83m formulations, *n* = 5 for control groups immunised with SPs or PBS) were immunised subcutaneously twice at 28-day intervals. The scheme of the study is presented in Figure 4. At 21 days after the second immunisation, all guinea pigs were subcutaneously challenged with *B. anthracis* strain 81/1 (2,500 spores per animal). At 20 days after the first challenge, all surviving guinea pigs were subjected to the second challenge with 25,000 spores of the same strain per animal. The animals were observed for 21 days after reinfection. Survival curves for the second challenge are presented in the figure. For each group, the number of challenged (Ñ) and surviving (*S*) animals are presented to the right. The results of statistical analysis of the differences in survival rates of the guinea pig immunisation groups are presented in Supplementary Table 3.

All guinea pigs that survived after the first challenge were subjected to reinfection with 25,000 spores of *B. anthracis* 81/1 strain (pXO1^+^, pXO2^+^) per animal. The survival curves for all animal groups after the second challenge are presented in Figure 7. The Gehan-Breslow-Wilcoxon test, with subsequent application of the Holm-Bonferroni method was used to analyse the differences in survival rate between all groups immunised with non-incubated rPA83m formulations and control groups. According to the test results, the survival rates of guinea pigs in the groups immunised with individually formulated rPA83m (group 1) or with rPA83m + SPs compositions (group 2) were both significantly higher than the survival rate of guinea pigs in the control group (the cumulative data from groups 7 and 8). At the same time, the group immunised with rPA83m + Al(OH)_3_ (group 3) did not significantly differ from the control group. Moreover, the survival rate of guinea pigs in group 2 was significantly higher than that of guinea pigs in group 3. The detailed results of the test are presented in Supplementary Table 3. The survival curves of the guinea pigs immunised with non-incubated rPA83m formulation were compared with those of guinea pigs immunised with corresponding incubated rPA83m formulation. No significant differences were revealed between the survivability rates of groups 1 and 4, groups 2 and 5, or groups 3 and 6.

## Discussion

The development of an effective, stable and safe anthrax vaccine remains a desirable goal. Previously, the authors designed an anthrax vaccine candidate based on rPA83m protein in composition with spherical particles (SPs) obtained from tobacco mosaic virus through its thermally-induced structural rearrangement (Ryabchevskaya et al., 2022). rPA83m is a modification of anthrax protective antigen (PA), with two major proteolytic sites being inactivated (^162^NSRKKR^167^ was substituted with ^162^QSSNKE^167^ at the furin-sensitive site and ^313^FF^314^ was deleted at the chymotrypsin-sensitive site) and two major deamidation-prone residues (Asn^713^ and Asn^719^) sites substituted by glutamines. The above-mentioned modifications were implemented in order to solve the low stability of rPA–the main problem associated with rPA-based vaccines. The stability of the rPA83m protein was preliminarily assessed and was demonstrated to be considerably greater than the stability of non-modified full-size rPA and this stability was increased further when it was a part of rPA83m + SPs compositions. During the current research, the detailed analysis of rPA83m stability in individual form and as a part of compositions with SPs was carried out under various temperature conditions. A study was made of the immunogenicity and the protectiveness of rPA83m + SPs compositions in comparison with individual rPA83m formulation and rPA83m adsorbed to aluminium hydroxide gel. The impact of storage on the observed properties of rPA83m formulations was also studied.

The evaluation of rPA83m and rPA83m + SPs compositions stability was carried out at +37°C (in order to model rapid protein ageing) for 33 days, at +25°C (a room temperature) for 231 days and at +4°C (a common vaccine storage temperature) for 276 days. The data obtained demonstrated that the stability of rPA83m at both +37°C and +25°C increases significantly when a part of rPA83m + SPs compositions, compared to that of individual rPA83m formulation. This finding fully supports previously performed qualitative evaluation (Ryabchevskaya et al., 2021, 2022). This enables the claim that using SPs as a stabiliser and a carrier for rPA83m is an effective way to supplement implemented modifications in the structure of rPA83m an approach to rPA stabilisation. The experiment at +4°C did not reveal any significant differences in the stability of individual rPA83m and rPA83m as a part of rPA83m + SPs compositions. At the same time, the relative amount of rPA83m remaining in all analysed samples throughout the 276-day incubation days did not fall below 0.6. Thus, the lack of a detectable impact of SPs on rPA83m stability in this experiment is likely to have been due to the incubation time chosen being insufficient to reveal it. However, the ability of rPA83m to remain in individually formulated samples in the relative amount of 0.6, even after 276 days of incubation at +4°C, is itself remarkable and indicates the effectiveness of the modifications implemented.

Previously, rPA83m was shown to be able to be adsorbed to SPs’ surface. The antigenic specificity of rPA83m was shown to remain after the adsorption. In particular, the ability to bind to PA-neutralising monoclonal antibodies remained (Ryabchevskaya et al., 2022). In the current research, the authors studied the immunogenicity of the vaccine candidate. The experiment was carried out using guinea pigs, which are a common model for the evaluation of anthrax vaccine effectiveness and are recommended by the United States Food and Drug administration for anthrax vaccines potency assessment (21 CFR 620.23). The immunogenicity of rPA83m + SPs compositions was compared to that of individually formulated rPA83m and rPA83m adsorbed to aluminium hydroxide gel, a common adjuvant for anthrax vaccines that has, however, been shown to decrease the stability of rPA (Wagner et al., 2012). Both rPA83m + SPs and rPA83m formulations were demonstrated to induce a reliably high anti-rPA83m antibodies titre and their immunogenicity did not differ. This indicates a high immunogenicity for the vaccine candidate and the lack of a decrease in rPA83m immunogenicity when adsorbed to SPs. The differences between anti-rPA83m antibodies titre elicited by rPA83m + Al(OH)_3_ and the titres elicited in control groups were not significant. Given this fact, aluminium hydroxide not only failed to act as an effective adjuvant for adsorbed rPA83m, but also tended to decrease its immunogenicity. This might partially be because the samples were exposed to storage at -20°C. Aluminium-based vaccines are categorised as being temperature-sensitive formulations, so their freezing may lead to the formation of aggregates of aluminium adjuvant and loss of a potency (Kurza̧tkowski et al., 2013). Thus, the temperature recommended by WHO for the storage of aluminium-based vaccines storage is +2 to +8°C (World Health Organization, 2015). In the current research, the storage temperature was -20°C, to equalise experimental conditions for all rPA83m formulations. Additionally, the possibility to store the vaccine in frozen form could be a significant advantage. Therefore, the revealed problems of low rPA83m + Al(OH)_3_ immunogenicity in this experiment, along with the previously demonstrated negative impact of Al(OH)_3_ on rPA stability, suggest that aluminium hydroxide might not be the most appropriate basis for a rPA83m-containing vaccine. Given the demonstrated ability of SPs to increase the stability of rPA83m, it seemed interesting to examine the immunogenicity of aged rPA83m formulations. The ageing of the samples was performed as a 27-day incubation at +37°C. The results were similar to those of the non-incubated samples, but, in addition, incubated rPA83m + SPs compositions were significantly more immunogenic, than incubated rPA83m + Al(OH)_3_ formulation. The latter finding does not speak in favour of aluminium hydroxide gel as a prospective adjuvant for rPA83m. Moreover, rPA83m + Al(OH)_3_ was the only rPA83m formulation that demonstrated a significant decrease in immunogenicity when stored, based on pairwise comparison of titre induced by corresponding incubated and non-incubated formulations. It is worth mentioning that, for incubated formulations, an F-test revealed a significantly lower variance for titre of anti-rPA83m antibodies induced by rPA83m + SPs formulation than one induced by individually formulated rPA83m. This might indicate higher levels of consistency in the immunogenicity of rPA83m + SPs after storage compared to individual rPA83m. Overall, the results obtained indicate that the vaccine candidate containing rPA83m and SPs effectively maintained immunogenic properties after storage. The neutralisation of anthrax bacteria by antibodies produced by B-cells is considered to be the main organism strategy to defeat anthrax (Quinn et al., 2004). However, it was found that T-cell responses to PA in naturally infected individuals encompassed Th1-regulatory cytokine IL-2 along with Th2-regulatory cytokines (IL-4, IL-9, IL-13) (Ingram et al., 2010), which evidences that Th1-mediated immunity could also be involved in anthrax defence. Presumably, inflammatory Th1 immune response may play a role by improving the pathogen clearance by macrophages. Previous studies suggest that SPs could mediate the stimulation of both Th1 and Th2 branches of adaptive immune response to the antigen administrated with them (Trifonova et al., 2017; Kurashova et al., 2020; Kovalenko et al., 2022). This enables to suppose that not only B-cells (and B-memory cells), but also both of Th1 and Th2 T-helpers with their corresponding memory cells specific to rPA83m are generated after vaccination with rPA83m + SPs compositions.

The evaluation of the immune response to the excipient vaccine components, that could be immunogenic by themselves, is important for vaccine candidate characterisation. The authors assessed the anti-SPs total IgG antibody titres in vaccinated animals’ sera. Both non-incubated and incubated rPA83m formulations induced reliably detectable IgG titres to SPs. However, those titres were significantly lower than anti-rPA83m titres induced by the corresponding vaccine formulations. This finding is consistent with previously collected data indicating that immunisation with antigen + SPs compositions leads to the induction of immune response predominantly to the antigen in the case of other SPs-based vaccine candidates (Trifonova et al., 2017; Evtushenko et al., 2020; Kovalenko et al., 2022). According to previously published studies, six times higher titres to the rubella antigen than to SPs has been detected after immunisation with rubella vaccine candidate (Trifonova et al., 2017), nine and 16-times higher titres to coronavirus antigens than to SPs have been induced by coronavirus vaccine candidate (Kovalenko et al., 2022) and a 47-fold predomination of antibodies to the target antigen have been revealed after immunisation with SPs compositions with ovalbumin (Evtushenko et al., 2020). In the present study, the total IgG titres to the target antigen was 150-times and 94-times higher than to the SPs in the case of immunisations with non-incubated and incubated rPA83m + SPs formulations, respectively. The crucial prevalence of anti-rPA83m antibodies determines the favourable immunogenicity profile of rPA83m + SPs compositions and confirms the possibility of applying SPs as a stabilising component for anthrax vaccine candidate.

The results of immunological experiments can only serve as supporting data in terms of the assessment of vaccine candidate effectiveness. Therefore, an experiment on vaccine candidate protectiveness was carried out using the immunised guinea pigs that were featured in the immunological experiment. The spores of fully virulent *B. anthracis* 81/1 strain were used for the challenge. Data on the LD_50_ for 81/1 strain vary in the published literature. There are several patented developments featuring the description of 81/1 strain properties. According to one of these sources, LD_50_ for this strain in guinea pigs might be 20 spores (Barkova et al., 2005). Another source claimed that it was in the range of 32–182 spores, with a mean of 76 (Buravtseva et al., 1999). Thus, in the current study, the authors considered an average LD_50_ for 81/1 strain in guinea pigs to be approximately 50. An experiment with various *B. anthracis* isolates carried out by Fellows et al. (2001) showed that the challenge with 100 LD_50_ Ames equivalents of fully virulent strains may lead to the death of animals in vaccinated groups (Fellows et al., 2001). Given these above-mentioned facts, it was decided that the first protectiveness assessment would be carried out with only 50 LD_50_ of 81/1 *B. anthracis* strain spores per animal (2,500 spores). This experiment found, however, that the mortality rates were low, even in control groups. This led to the decision to perform an additional challenge, with 25,000 spores per animal (approximately 500 LD_50_) with the survivors of the first experiment. The results obtained demonstrated the protectiveness of both individually formulated rPA83m and rPA83m + SPs compositions, and their protective properties did not differ significantly. This enables to claim that SPs do not have a negative impact on the protectiveness of rPA83m, while being able to stabilise it, as has been shown in this paper. At the same time, rPA83m + Al(OH)_3_ did not provide an effective level of protectiveness, and the protective properties of rPA83m + Al(OH)_3_ were significantly lower than those of rPA83m + SPs compositions. The impact of ageing on the protectiveness of rPA83m formulations was also evaluated. Twenty seven-day incubation at +37°C resulted in no decrease in the protectiveness of any studied formulations. According to the stability experiments data, the ageing performed should result in 5–10-fold decrease in the amount of individually formulated rPA83m. The latter facts suggest that the dosage of rPA83m used for immunisation in each formulation was excessive. Further studies will focus on decreasing the antigen dosage, which should be an indisputable advantage of a vaccine candidate.

## Limitation of the present study

The present study mainly focuses on the assessment of the vaccine candidate effectiveness in animals and has various strengths and limitations. In terms of strengths, the protectiveness against fully virulent *B. anthracis* strain was evaluated in guinea pigs. Limitation of the study is the fact that the impact of SPs on rPA83m stability has to be additionally supported by the data obtained using other analytical methods. Importantly, the study of protectiveness conducted with the lower antigen dosage may enable to reveal the SPs impact on rPA83m stability and is primary subject of our further work. Possibly, not only stability but also immunogenicity of rPA83m might be improved by SPs in case the amount of rPA83m per dose will be considerably decreased because SPs were previously shown to be able to act as an adjuvant.

## Data availability statement

The original contributions presented in this study are included in the article/Supplementary material, further inquiries can be directed to the corresponding author/s.

## Ethics statement

The animal study was reviewed and approved by the State Research Center for Applied Microbiology and Biotechnology Bioethics Committee (Permit No: VP 2021/4 dated 16.06.2021).

## Author contributions

OVK supervised the study. OVK, EE, NN, VT, OAK, ER, and DG conceived and designed the experiments. NN and MA performed virus propagation and SPs production. DG, ER, and MA accumulated recombinant protein. VT, IB, TK, ER, and EE performed immunisation and blood collection. VT, IB, and TK carried out animal challenge. DG and ER performed the stability study and IgG titres assessment. DG and EE carried out data curation, statistical analysis, and results visualization. DG and ER wrote the initial draft of the manuscript with comments and editing provided by all authors. All authors contributed to the article and approved the submitted version.

## Supplementary material

Supplementary Figure 1 The dynamic of weight of guinea pigs in groups immunised with rPA83m formulations and in control groups immunised with SPs or PBS. Dynamic of guinea pigs’ weight in the group immunised with non-incubated rPA83m **(A)**, non-incubated rPA83m + SPs **(B)**, non-incubated rPA83m + Al(OH)_3_ **(C)**, incubated rPA83m **(D)**, incubated rPA83m + SPs **(E)**, incubated rPA83m + Al(OH)_3_ **(F)**, or SPs **(G)** comparing to group immunised with PBS. Incubated formulations marked with “inc.” were incubated at +37°C for 27 days. Groups of guinea pigs (*n* = 10 for groups immunised with rPA83m formulations, *n* = 5 for control groups immunised with SPs or PBS) were immunised subcutaneously twice at 28-days intervals. The scheme of the study is presented in Figure 4. The weight of immunised guinea pigs was analysed at 3-day intervals. The mean of guinea pigs’ weight for each group was used for graph plotting. Bars represent 95% CI.

Supplementary Figure 2 Protectiveness of rPA83m formulations in guinea pigs after first *Bacillus anthracis* strain 81/1 anthrax spore challenge. **(A)** Protectiveness of non-incubated rPA83m formulations. Figure represents survival curves of guinea pig groups immunised with non-incubated formulations of rPA83m, rPA83m + SPs, rPA83 + Al(OH)_3_, as well as SPs or PBS. **(B)** Protectiveness of incubated rPA83m formulations. Figure represents survival curves of guinea pig groups immunised with incubated formulations of rPA83m, rPA83m + SPs, rPA83 + Al(OH)_3_, as well as SPs or PBS. Incubated formulations marked with “inc.” were incubated at +37°C for 27 days. Groups of guinea pigs (*n* = 10 for groups immunised with rPA83m formulations, *n* = 5 for control groups immunised with SPs or PBs) were immunised subcutaneously twice at 28-day intervals. The scheme of the study is presented in Figure 4. In 21 days after the second immunisation, all guinea pigs were subcutaneously challenged with *B. anthracis* strain 81/1 (2,500 spores per animal). The animals were observed for 20 days after challenge. Survival curves are presented in the figure.
