# Supplementary figures and images for: New formulation of a recombinant anthrax vaccine stabilised with structurally modified plant viruses
Source: Front Microbiol. 2022 Sep 9;13:1003969. doi: 10.3389/fmicb.2022.1003969 (PMC9501872; doi:10.3389/fmicb.2022.1003969)

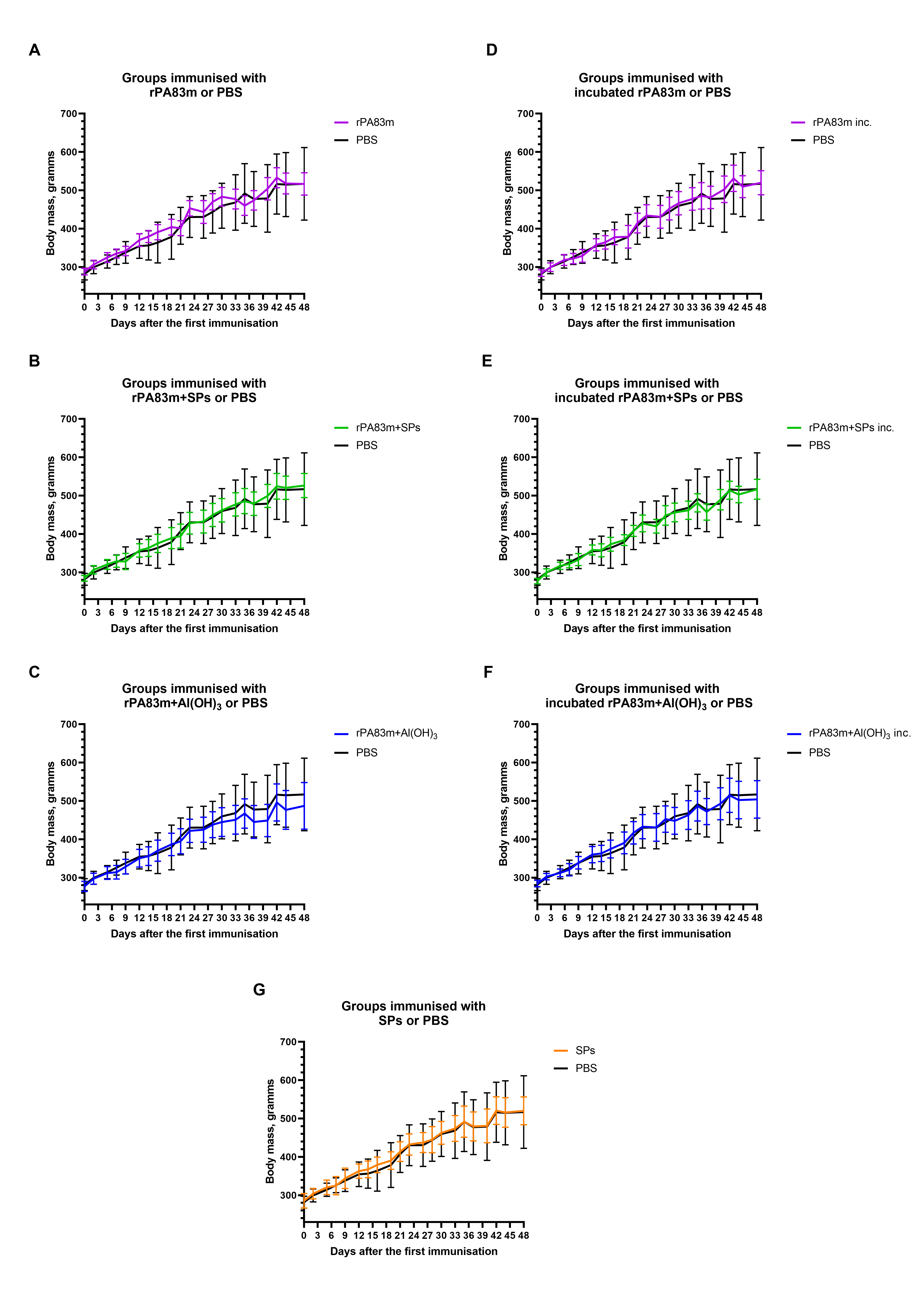

Supplement: Supplementary Figure 1 — The dynamic of weight of guinea pigs in groups immunised with rPA83m formulations and in control groups immunised with SPs or PBS. Dynamic of guinea pigs’ weight in the group immunised with non-incubated rPA83m (A), non-incubated rPA83m + SPs (B), non-incubated rPA83m + Al(OH)3 (C), incubated rPA83m (D), incubated rPA83m + SPs (E), incubated rPA83m + Al(OH)3 (F), or SPs (G) comparing to group immunised with PBS. Incubated formulations marked with “inc.” were incubated at +37°C for 27 days. Groups of guinea pigs (n = 10 for groups immunised with rPA83m formulations, n = 5 for control groups immunised with SPs or PBS) were immunised subcutaneously twice at 28-days intervals. The scheme of the study is presented in Figure 4. The weight of immunised guinea pigs was analysed at 3-day intervals. The mean of guinea pigs’ weight for each group was used for graph plotting. Bars represent 95% CI. [file Image_1.TIF]

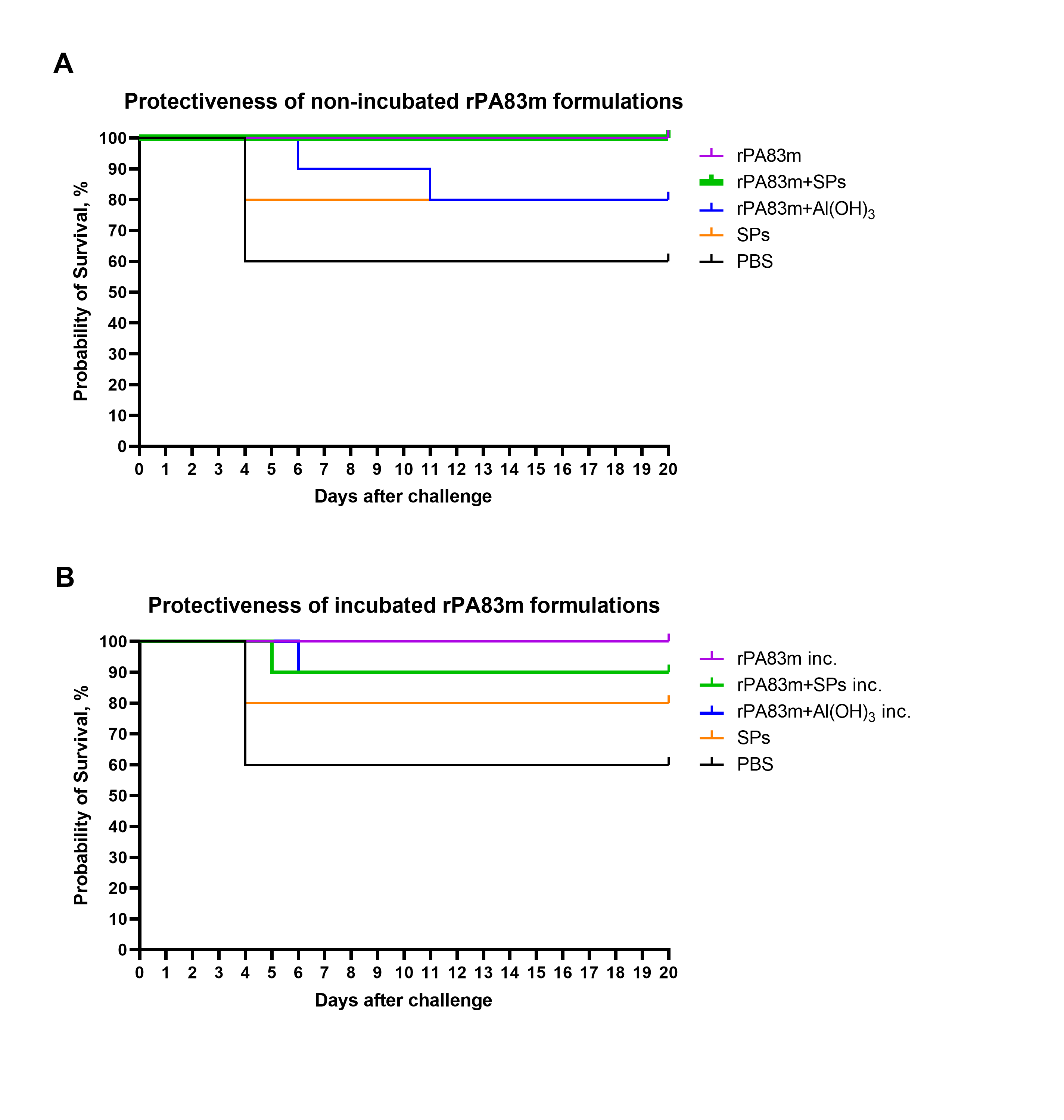

Supplement: Supplementary Figure 2 — Protectiveness of rPA83m formulations in guinea pigs after first Bacillus anthracis strain 81/1 anthrax spore challenge. (A) Protectiveness of non-incubated rPA83m formulations. Figure represents survival curves of guinea pig groups immunised with non-incubated formulations of rPA83m, rPA83m + SPs, rPA83 + Al(OH)3, as well as SPs or PBS. (B) Protectiveness of incubated rPA83m formulations. Figure represents survival curves of guinea pig groups immunised with incubated formulations of rPA83m, rPA83m + SPs, rPA83 + Al(OH)3, as well as SPs or PBS. Incubated formulations marked with “inc.” were incubated at +37°C for 27 days. Groups of guinea pigs (n = 10 for groups immunised with rPA83m formulations, n = 5 for control groups immunised with SPs or PBs) were immunised subcutaneously twice at 28-day intervals. The scheme of the study is presented in Figure 4. In 21 days after the second immunisation, all guinea pigs were subcutaneously challenged with B. anthracis strain 81/1 (2,500 spores per animal). The animals were observed for 20 days after challenge. Survival curves are presented in the figure. [file Image_2.TIF]
