# Supplementary material for: New formulation of a recombinant anthrax vaccine stabilised with structurally modified plant viruses
Source: Front Microbiol. 2022 Sep 9;13:1003969. doi: 10.3389/fmicb.2022.1003969 (PMC9501872; doi:10.3389/fmicb.2022.1003969)
Supplement: Supplementary file 3 [file Table_1.docx]

| **Total IgG to rPA83m** | | | | | | | | |
| --- | --- | --- | --- | --- | --- | --- | --- | --- |
| **Immunisation group** | **Identification number of guinea pig** | **Titer** | **log_10_titer** | **Immunisation group** | **Identification number of guinea pig** | **Titer** | **log_10_titer** |  |
| **1 (rPA83m)** | 1.1 | 4,11x10^6^ | 6,61 | **4 (rPA83m incubated)** | 4.1 | 2,13x10^6^ | 6,33 |  |
|  | 1.2 | 4,23x10^6^ | 6,63 |  | 4.2 | 1,83x10^6^ | 6,26 |  |
|  | 1.3 | 5,23x10^6^ | 6,72 |  | 4.3 | 2,76x10^6^ | 6,44 |  |
|  | 1.4 | 1,42x10^7^ | 7,15 |  | 4.4 | 4,70x10^5^ | 5,67 |  |
|  | 1.5 | 6,15x10^6^ | 6,79 |  | 4.5 | 1,23x10^7^ | 7,09 |  |
|  | 1.6 | 8,09x10^6^ | 6,91 |  | 4.6 | 8,96x10^6^ | 6,95 |  |
|  | 1.7 | 2,95x10^6^ | 6,47 |  | 4.7 | 2,34x10^6^ | 6,37 |  |
|  | 1.8 | 1,11x10^7^ | 7,04 |  | 4.8 | 3,52x10^5^ | 5,55 |  |
|  | 1.9 | 1,68x10^6^ | 6,22 |  | 4.9 | 3,67x10^6^ | 6,56 |  |
|  | 1.10 | 3,38x10^6^ | 6,53 |  | 4.10 | 6,04x10^5^ | 5,78 |  |
|  | Median | 4,73x10^6^ | 6,67 |  | Median | 2,23x10^6^ | 6,35 |  |
| **2 (rPA83m+SPs)** | 2.1 | 1,15x10^7^ | 7,06 | **5 (rPA83m+SPs incubated)** | 5.1 | 1,65x10^6^ | 6,22 |  |
|  | 2.2 | 8,23x10^6^ | 6,92 |  | 5.2 | 6,29x10^6^ | 6,80 |  |
|  | 2.3 | 1,79x10^6^ | 6,25 |  | 5.3 | 1,06x10^6^ | 6,03 |  |
|  | 2.4 | 1,01x10^7^ | 7,01 |  | 5.4 | 2,48x10^6^ | 6,39 |  |
|  | 2.5 | 1,40x10^7^ | 7,15 |  | 5.5 | 4,67x10^6^ | 6,67 |  |
|  | 2.6 | 9,66x10^6^ | 6,98 |  | 5.6 | 1,95x10^6^ | 6,29 |  |
|  | 2.7 | 1,71x10^6^ | 6,23 |  | 5.7 | 1,78x10^6^ | 6,25 |  |
|  | 2.8 | 4,51x10^6^ | 6,65 |  | 5.8 | 5,38x10^6^ | 6,73 |  |
|  | 2.9 | 5,66x10^6^ | 6,75 |  | 5.9 | 6,05x10^6^ | 6,78 |  |
|  | 2.10 | 3,73x10^6^ | 6,57 |  | 5.10 | 1,11x10^6^ | 6,05 |  |
|  | Median | 6,94x10^6^ | 6,84 |  | Median | 2,21x10^6^ | 6,35 |  |
| **3 (rPA83m+Al(OH)_3_)** | 3.1 | 1,05x10^6^ | 6,02 | **6 (rPA83m+Al(OH)_3_ incubated)** | 6.1 | 4,14x10^3^ | 3,62 |  |
|  | 3.2 | 1,48x10^6^ | 6,17 |  | 6.2 | 2,09x10^5^ | 5,32 |  |
|  | 3.3 | 2,60x10^5^ | 5,42 |  | 6.3 | 2,46x10^5^ | 5,39 |  |
|  | 3.4 | 1,40x10^6^ | 6,15 |  | 6.4 | 3,70x10^3^ | 3,57 |  |
|  | 3.5 | 4,38x10^6^ | 6,64 |  | 6.5 | 2,52x10^5^ | 5,40 |  |
|  | 3.6 | 8,69x10^5^ | 5,94 |  | 6.6 | 2,23x10^5^ | 5,35 |  |
|  | 3.7 | 3,09x10^5^ | 5,49 |  | 6.7 | 8,27x10^4^ | 4,92 |  |
|  | 3.8 | 1,57x10^6^ | 6,20 |  | 6.8 | 1,73x10^6^ | 6,24 |  |
|  | 3.9 | 1,84x10^6^ | 6,27 |  | 6.9 | 5,88x10^5^ | 5,77 |  |
|  | 3.10 | 4,58x10^5^ | 5,66 |  | 6.10 | 1,49x10^4^ | 4,17 |  |
|  | Median | 1,22x10^6^ | 6,09 |  | Median | 2,16x10^5^ | 5,33 |  |
| **7 (SPs)** | 7.1 | 9,40x10^2^ | 2,97 | **8 (PBS)** | 8.1 | 2,78x10^3^ | 3,44 |  |
|  | 7.2 | 2,32x10^3^ | 3,37 |  | 8.2 | 5,38x10^3^ | 3,73 |  |
|  | 7.3 | 3,15x10^3^ | 3,50 |  | 8.3 | 4,22x10^3^ | 3,63 |  |
|  | 7.4 | 1,22x10^3^ | 3,09 |  | 8.4 | 1,33x10^3^ | 3,12 |  |
|  | 7.5 | 1,17x10^4^ | 4,07 |  | 8.5 | 8,75x10^2^ | 2,94 |  |
|  | Median | 1,80x10^3^ | 3,26 |  | Median | 1,77x10^3^ | 3,25 |  |

**SUPPLEMENTARY TABLE 1 |** Total IgG titers to rPA83m in blood sera of guinea pigs from groups immunised with rPA83m formulations and control groups immunised with SPs or PBS. Groups of guinea pigs were immunised subcutaneously twice at 28-day intervals. The scheme of the study is presented in **Figure 4**. Blood was collected from the marginal ear vein 20 days after the second immunisation. Sera titers were evaluated using indirect ELISA (antigen concentration on microplate – 10 µg/ml).
