# Supplementary material for: New formulation of a recombinant anthrax vaccine stabilised with structurally modified plant viruses
Source: Front Microbiol. 2022 Sep 9;13:1003969. doi: 10.3389/fmicb.2022.1003969 (PMC9501872; doi:10.3389/fmicb.2022.1003969)
Supplement: Supplementary file 4 [file Table_2.docx]

| **Total IgG to SPs** | | | | | | | |
| --- | --- | --- | --- | --- | --- | --- | --- |
| **Immunisation group** | **Identification number of guinea pig** | **Titer** | **log_10_titer** | **Immunisation group** | **Identification number of guinea pig** | **Titer** | **log_10_titer** |
| **2 (rPA83m+SPs)** | 2.1 | 7.28x10^3^ | 3.86 | **5 (rPA83m+SPs incubated)** | 5.1 | 6.90x10^3^ | 3.84 |
|  | 2.2 | 5.15x10^4^ | 4.71 |  | 5.2 | 1.36x10^4^ | 4.13 |
|  | 2.3 | 5.74x10^3^ | 3.76 |  | 5.3 | 2.02x10^4^ | 4.31 |
|  | 2.4 | 4.40x10^4^ | 4.64 |  | 5.4 | 3.64x10^4^ | 4.56 |
|  | 2.5 | 4.92x10^4^ | 4.69 |  | 5.5 | 1.52x10^4^ | 4.18 |
|  | 2.6 | 5.03x10^4^ | 4.70 |  | 5.6 | 1.01x10^5^ | 5.00 |
|  | 2.7 | 4.85x10^4^ | 4.69 |  | 5.7 | 4.86x10^4^ | 4.69 |
|  | 2.8 | 1.17x10^4^ | 4.07 |  | 5.8 | 2.73x10^4^ | 4.44 |
|  | 2.9 | 5.75x10^3^ | 3.76 |  | 5.9 | 6.93x10^3^ | 3.84 |
|  | 2.10 | 8.37x10^4^ | 4.92 |  | 5.10 | 2.69x10^4^ | 4.43 |
|  | Median | 4.63x10^4^ | 4.67 |  | Median | 2.35x10^4^ | 4.37 |
| **7 (SPs)** | 7.1 | 5.10x10^4^ | 4.71 | **8 (PBS)** | 8.1 | 6.41x10^3^ | 3.81 |
|  | 7.2 | 6.20x10^4^ | 4.79 |  | 8.2 | 5.52x10^3^ | 3.74 |
|  | 7.3 | 2.06x10^4^ | 4.31 |  | 8.3 | 5.41x10^3^ | 3.73 |
|  | 7.4 | 3.19x10^4^ | 4.50 |  | 8.4 | 6.82x10^3^ | 3.83 |
|  | 7.5 | 1.24x10^4^ | 4.09 |  | 8.5 | 4.44x10^3^ | 3.65 |
|  | Median | 3.19x10^4^ | 4.50 |  | Median | 5.52x10^3^ | 3.74 |

**SUPPLEMENTARY TABLE 2 |** Total IgG titers to SPs in blood sera of guinea pigs from groups immunised with rPA83m+SPs formulations and control groups immunised with SPs or PBS. Groups of guinea pigs were immunised subcutaneously twice at 28-day intervals. The scheme of the study is presented in **Figure 4**. Blood was collected from the marginal ear vein 20 days after the second immunisation. Sera titers were evaluated using indirect ELISA (antigen concentration on microplate – 10 µg/ml).
