# Supplementary material for: New formulation of a recombinant anthrax vaccine stabilised with structurally modified plant viruses
Source: Front Microbiol. 2022 Sep 9;13:1003969. doi: 10.3389/fmicb.2022.1003969 (PMC9501872; doi:10.3389/fmicb.2022.1003969)
Supplement: Supplementary file 5 [file Table_3.docx]

| Guinea pig immunisation group | rPA83m | rPA83m+SPs | rPA83m+Al(OH)_3_ | Control  (SPs and PBS groups) |
| --- | --- | --- | --- | --- |
| rPA83m |  | 0.1468 | 0.0325 | 0.0079 |
| rPA83m+SPs | 0.1468 |  | 0.004 | 0.002 |
| rPA83m+Al(OH)_3_ | 0.0325 | 0.004 |  | 0.2596 |
| Control (SPs and PBS groups) | 0.0079 | 0.002 | 0.2596 |  |

**SUPPLEMENTARY TABLE 3 |** Statistical analysis of the differences in survival rates of guinea pigs immunised with various non-incubated rPA83m formulations after the spore challenge with 25,000 *B. anthracis* 81/1 strain spores per animal. Groups of guinea pigs (n = 10 for groups immunised with rPA83m formulations, n = 5 for control groups immunised with SPs or PBS) were immunised subcutaneously twice at 28-day intervals. The scheme of the study is presented in **Figure 4**. At 21 days after the second immunisation, all guinea pigs were subcutaneously challenged with *B. anthracis* strain 81/1 (2,500 spores per animal). At 20 days after the first challenge all survived guinea pigs were subjected to the second challenge with 25,000 spores of the same strain per animal. The animals were observed for 21 days after reinfection. For comparison of the survival rate between different groups the Gehan-Breslow-Wilcoxon test was used and the Holm-Bonferroni method was applied for adjusting p-values for multiple comparisons. *P-*values are presented in the figure. *P-*values between groups with statistically significant differences in the survival rate after the Holm-Bonferroni method application are highlighted in green. *P-*values between groups without statistically significant differences are highlighted in red.
